# Supplementary material for: Number of transurethral procedures after non-muscle-invasive bladder cancer and survival in causes other than bladder cancer
Source: PLoS One. 2022 Sep 23;17(9):e0274859. doi: 10.1371/journal.pone.0274859 (PMC9506622; doi:10.1371/journal.pone.0274859)
Supplement: Attachment — Submitted filename: Response to reviewers.docx [file pone.0274859.s001.docx]

PONE-D-22-17365

Number of transurethral procedures after non-muscle-invasive bladder cancer and survival in causes other than bladder cancer

PLOS ONE

Dear Dr Huang,

Thank you for a rapid and concise management of our manuscript. Please find our response below.

Journal Requirements:

When submitting your revision, we need you to address these additional requirements.

1. Please ensure that your manuscript meets PLOS ONE's style requirements, including those for file naming. The PLOS ONE style templates can be found at 
<https://journals.plos.org/plosone/s/file?id=wjVg/PLOSOne_formatting_sample_main_body.pdf> and 
<https://journals.plos.org/plosone/s/file?id=ba62/PLOSOne_formatting_sample_title_authors_affiliations.pdf>.

**Answer**: We have now formatted the manuscript according to the templates and hope we have interpreted the instructions correctly.

2. Please provide additional details regarding participant consent. In the ethics statement in the Methods and online submission information, please ensure that you have specified what type you obtained (for instance, written or verbal, and if verbal, how it was documented and witnessed). If your study included minors, state whether you obtained consent from parents or guardians. If the need for consent was waived by the ethics committee, please include this information.

**Answer:** The Research Ethics Board of Uppsala University, Sweden (Ref no. 2015/277) and Swedish Ethical Review Authority (Ref no. 2019-03574 and 2020-05123) waived the informed consent requirement for this study. *This is now clarified in the manuscript.*

3. We note that the grant information you provided in the ‘Funding Information’ and ‘Financial Disclosure’ sections do not match.

When you resubmit, please ensure that you provide the correct grant numbers for the awards you received for your study in the ‘Funding Information’ section.

**Answer:** they did not match because the statement in the manuscript did not specify who the respective PI:s were for each grant – the grant numbers were however correct. We have understood the instructions so that the funding statement should **not** be included in the manuscript and only in the on-line form. Therefore, the statement is excluded from the manuscript. We also tried to make the funding information clearer:

*This work was supported by the Swedish Cancer Society (grant numbers CAN 2019/62 (Holmberg) and CAN 2020/0709 (Liedberg), Swedish Research Council (2021-00859) (Liedberg), Lund Medical Faculty (ALF) (Liedberg), and Skåne County Council’s Research and Development Foundation (Liedberg). The funding sources had no role in the study design, data analyses, interpretation of the results, or writing of the manuscript. Swedish Cancer Society: Cancerfonden.se; Swedish Research Council: vr.se; Lund Medical Faculty: lu.se; Skåne County: Skane.se*

4. In your Data Availability statement, you have not specified where the minimal data set underlying the results described in your manuscript can be found. PLOS defines a study's minimal data set as the underlying data used to reach the conclusions drawn in the manuscript and any additional data required to replicate the reported study findings in their entirety. All PLOS journals require that the minimal data set be made fully available. For more information about our data policy, please see <http://journals.plos.org/plosone/s/data-availability>.

"Upon re-submitting your revised manuscript, please upload your study’s minimal underlying data set as either Supporting Information files or to a stable, public repository and include the relevant URLs, DOIs, or accession numbers within your revised cover letter. For a list of acceptable repositories, please see <http://journals.plos.org/plosone/s/data-availability#loc-recommended-repositories>. Any potentially identifying patient information must be fully anonymized.

Important: If there are ethical or legal restrictions to sharing your data publicly, please explain these restrictions in detail. Please see our guidelines for more information on what we consider unacceptable restrictions to publicly sharing data: <http://journals.plos.org/plosone/s/data-availability#loc-unacceptable-data-access-restrictions>. Note that it is not acceptable for the authors to be the sole named individuals responsible for ensuring data access.

We will update your Data Availability statement to reflect the information you provide in your cover letter.

**Answer:** We are aware that this is a question often raised re Swedish register-based research and we are sorry that we were too brief in our statement. Please find below a more detailed explanation:

*Data used in the present study was extracted from the research database BladderBaSe, which is based on the Swedish National Registry of Urinary Bladder Cancer (SNRUBC) and linkage to several national health-data registers. The data cannot be shared publicly because the individual-level data contain potentially identifying and sensitive patient information and cannot be published due to legislation and ethical review restrictions (*[*https://etikprovningsmyndigheten.se*](https://eur03.safelinks.protection.outlook.com/?url=https%3A%2F%2Fetikprovningsmyndigheten.se%2F&data=05%7C01%7Clars.holmberg%40kcl.ac.uk%7Ce80dc6e0935e40976a2508da802dfd3a%7C8370cf1416f34c16b83c724071654356%7C0%7C0%7C637963232858180432%7CUnknown%7CTWFpbGZsb3d8eyJWIjoiMC4wLjAwMDAiLCJQIjoiV2luMzIiLCJBTiI6Ik1haWwiLCJXVCI6Mn0%3D%7C3000%7C%7C%7C&sdata=UdaLbu%2FRMJ0gR77mhhXAe%2BORRQx76HQc%2BZvmBTqi5fU%3D&reserved=0)*). Use of the data from national health-data registers is further restricted by the Swedish Board of Health and Welfare (*[*https://www.socialstyrelsen.se/en/*](https://eur03.safelinks.protection.outlook.com/?url=https%3A%2F%2Fwww.socialstyrelsen.se%2Fen%2F&data=05%7C01%7Clars.holmberg%40kcl.ac.uk%7Ce80dc6e0935e40976a2508da802dfd3a%7C8370cf1416f34c16b83c724071654356%7C0%7C0%7C637963232858180432%7CUnknown%7CTWFpbGZsb3d8eyJWIjoiMC4wLjAwMDAiLCJQIjoiV2luMzIiLCJBTiI6Ik1haWwiLCJXVCI6Mn0%3D%7C3000%7C%7C%7C&sdata=mS5f%2FLIBjy%2BWzLa0yp7MkYwmaT3NTllbQMgldgoSOu8%3D&reserved=0)*) and Statistics Sweden (*[*https://www.scb.se/en/*](https://eur03.safelinks.protection.outlook.com/?url=https%3A%2F%2Fwww.scb.se%2Fen%2F&data=05%7C01%7Clars.holmberg%40kcl.ac.uk%7Ce80dc6e0935e40976a2508da802dfd3a%7C8370cf1416f34c16b83c724071654356%7C0%7C0%7C637963232858180432%7CUnknown%7CTWFpbGZsb3d8eyJWIjoiMC4wLjAwMDAiLCJQIjoiV2luMzIiLCJBTiI6Ik1haWwiLCJXVCI6Mn0%3D%7C3000%7C%7C%7C&sdata=9s6q4NYxpPSAkdqidTWacTiwNZ8NAcf9iOIzAFUBxpw%3D&reserved=0)*) which are Government Agencies providing access to the linked healthcare registers.*

*The data in in BladderBaSe is partly available in annual reports from the Swedish National Registry of Urinary Bladder Cancer (SNRUBC) and are also accessible online at* [*https://statistik.incanet.se/urinblasecancer/*](https://eur03.safelinks.protection.outlook.com/?url=https%3A%2F%2Fstatistik.incanet.se%2Furinblasecancer%2F&data=05%7C01%7Clars.holmberg%40kcl.ac.uk%7C9f4cc0ad646e43d074b808da7f9d90f3%7C8370cf1416f34c16b83c724071654356%7C0%7C0%7C637962612244905403%7CUnknown%7CTWFpbGZsb3d8eyJWIjoiMC4wLjAwMDAiLCJQIjoiV2luMzIiLCJBTiI6Ik1haWwiLCJXVCI6Mn0%3D%7C3000%7C%7C%7C&sdata=To8JKiTDZcNFx8FTL44nSYPgwUJYsIuGzjgUOk7D0sw%3D&reserved=0)*. Researchers can propose and apply for data and studies in BladderBaSe or SNRUBC using a standardized form. After approved application, the project data administrators can upload study-specific files with selected variables to a server for statistical analysis through remote access.*

5. Please review your reference list to ensure that it is complete and correct. If you have cited papers that have been retracted, please include the rationale for doing so in the manuscript text, or remove these references and replace them with relevant current references. Any changes to the reference list should be mentioned in the rebuttal letter that accompanies your revised manuscript. If you need to cite a retracted article, indicate the article’s retracted status in the References list and also include a citation and full reference for the retraction notice.

**Answer:** These papers were accessed at PubMed the last week and there is no indication that any of them were retracted.

Additional Editor Comments:

Dear Prof. Holmberg,

We appreciate your submission to PLoS ONE. In addition to the reviewers’ comments, I would like to bring up a couple of points required your clarifications:

1. Firstly, I feel confused as regards the comparison cohort was matched on what variables exactly, as there were inconsistent statements in your paper --

(a). P. 5, lines 103-104, “Controls were matched for sex, year of birth and county of residence”.
(b). P. 7, lines 169-170, “… the comparison cohort, matched for age, sex and county. We adjusted the models for prognostic group, CCI and educational level but not for the matching factors”.
(c). P. 8, lines 178-179, “These analyses were adjusted for prognostic group, CCI, educational level and age”.

Based on the statistics in Table 1, I assume the matched variables were sex, age, and education.

**Answer:** The matching between the NMIBC-patients and their comparison cohort was for sex, year of birth and county of residence. *We thank you for indicating that we did not word this consistently and have now done so in the manuscript*.

The analyses mentioned under (c) above were not done on the matched dataset; they did not include the comparison cohort which *we now have underlined more specifically* in the manuscript. Thus, these analyses did not include matching, but adjustment for prognostic group, CCI, educational level and age.

*We have checked the manuscript so that we use “matching” referring to the matching between NMIBC patients and the comparison cohort and “adjusting” for when models include co-variates that potentially could be confounders.*

2. Furthermore, concerning some 95% CI statistics:

(a). P. 12, lines 240-241, “… however with lower unadjusted HRs than for overall mortality varying from 1.04 (95% CI 1.00-1.07)”.
(b). P. 12, line 232, “… the absolute difference at ten years was 1.4% (95% CI 0.7%-2.1%)”.

If the confidence interval crosses 1, then it implies that the HR is not statistically significant (i.e., there is no difference between arms of the study).

**Answer**: Regarding (a): we have followed the convention that if the confidence interval does not encompass 1 – but only “touches” 1 - there is a sign that the null-hypothesis is statistically refuted. (In fact, the confidence interval here was 1.0028-1.07).

Regarding (b): this estimate refers to the absolute and not the relative difference and thus the point of interest in using the confidence interval as an indicator of statistical significance is 0% here. *We have further stressed this in the manuscript by pointing out that the 1.4 represent percentage points of difference and does not indicate a relative measure.*

**Reviewer #1**: the authors reported their experience on Number of transurethral procedures after non-muscle-invasive bladder cancer and survival in causes other than bladder cancer.
the research is well pconducted and well presented.
Anymway, I suggest some rivisions:
- please state the sample size calculation

**Answer**: To attain the highest statistical precision possible given the patients included in BladderBase, all available information in the register was used. *This is now clarified in the manuscript with an example of the resulting statistical power*: With 2000 events, a hazard ratio as 1.2 is detectable under standard assumptions (80 % power, two-sided 5 % significance level).

- when possible, perform a flow chart on included/exluded patients

**Answer:** There were no exclusions or losses to follow-up. Thus, we deem a flow-chart would be little informative, but are willing to add one if the editor so require. *We have clarified in the manuscript that all patients meeting the inclusion criteria were followed.*

- were patients consecutive?

**Answer:** Yes, the treating facilities enter patients consecutively into the SNRUBC, which is also checked against the National Cancer Register. This is now clarified in the manuscript.

- in my opinion, it is better to evaluate the risk classification rather than the grade alone of bladder cancer

**Answer:** We agree that it might have been informative to use the EAU risk scores for progression in our stratified analyses. This was not possible due to that the SNRUBC only recently included all the information needed for that classification and too few patients could be classified according to the EAU criteria. However, we used risk strata that underpins the clinical decision-making for Swedish NMIBC patients. *This issue is now discussed under “Strengths and limitations”.*

**Reviewer #2**: The Authors investigated the overall and disease-specific risk of death in patients with NMIBC
28 compared to a background population sample. The manuscript is well written, with a good statistical analysis and explore an interesting topic. My compliments to the Authors.

We hope that our revision addresses all the concerns raised.

With kind regards

Lars Holmberg

Prof emeritus Uppsala University and King’s College London.
